# A case of bullous pemphigoid showing antigenic competition‐like phenomenon

**DOI:** 10.1002/ccr3.2981

**Published:** 2020-06-17

**Authors:** Masahiro Fukuda, Yoshimasa Nobeyama, Hiroko Sekiyama, Akihiko Asahina

**Affiliations:** ^1^ Department of Dermatology The Jikei University School of Medicine Tokyo Japan

**Keywords:** antigenic competition, autoimmune bullous disease, bullous pemphigoid, current eruption, pre‐existing eruption

## Abstract

Antigenic competition in the skin is a phenomenon in which the current dermatitis is distributed away from the area of previously existing dermatitis. Bullous pemphigoid may present such phenomenon, even if the responsible antigen was the same.

## Graphical abstract

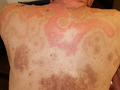

## 1 INTRODUCTION

Antigenic competition in the skin is a phenomenon in which the current dermatitis is distributed away from the area of previously existing dermatitis.^1^ We encountered a case of bullous pemphigoid (BP) showing this curious phenomenon.

## 2 CASE REPORT

**FIGURE 1:**
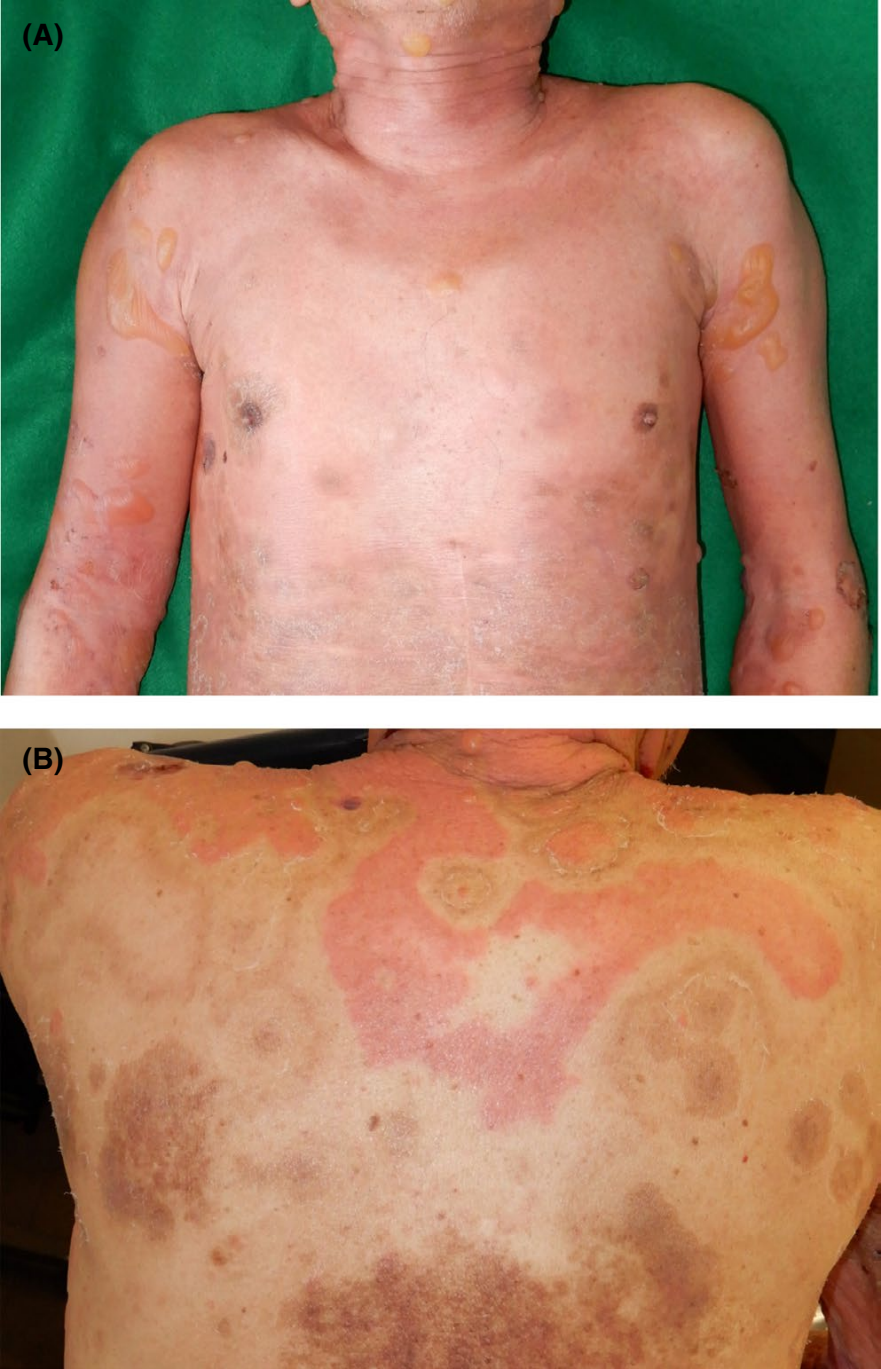
Clinical findings. A, Clinical manifestations on the trunk and upper limbs. Erythema, bullae, erosions, and pigmentation are observed. B, On the back, erythema is distributed away from the pigmented areas corresponding to the inactive lesion. An intact zone of about 2 cm in width clearly separates active and inactive lesions

**FIGURE 2:**
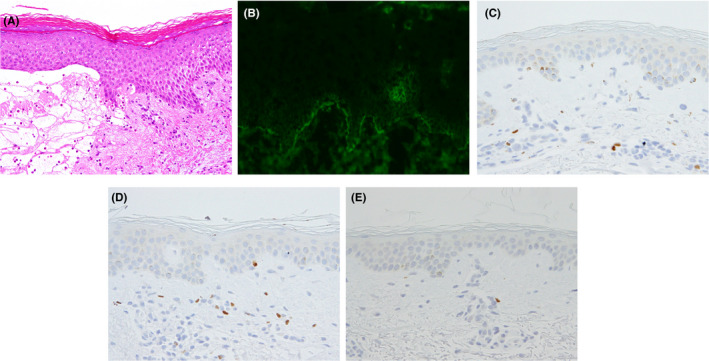
Histopathological findings. A, Histopathological findings of the erythema and bullae (hematoxylin‐eosin stain, ×40). Subepidermal blistering accompanied by eosinophilic infiltration is evident. B, Findings of direct immunofluorescent assay (×200). Immunoglobulin G is deposited in the basement membrane zone. C, Immunohistochemical analysis in the active lesion using anti‐FoxP3 antibody (Thermo Fisher Scientific). A few positive cells are shown (×400). D, Immunohistochemical analysis in the inactive lesion using anti‐FoxP3 antibody. Several positive cells are shown (×400). E, Immunohistochemical analysis in the intact skin using anti‐FoxP3 antibody. Few positive cells are shown (×400)

A 77‐year‐old Japanese man was referred to us with a 1‐year history of pruritic erythema, bulla, and erosion on the head/neck, trunk, and extremities (Figure 1A). The patient had already been diagnosed with BP in the previous clinic based on a high titer of serum anti‐BP180 NC16a domain antibodies. On the back, the erythema was found to be distributed away from the pigmented areas corresponding to the inactive lesion (Figure 1B). An intact zone of about 2 cm in width clearly separated active erythematous lesions from inactive pigmented lesions. Cross‐linked enzyme aggregate assay indicated > 1000 U/mL of serum anti‐BP180 NC16a domain antibodies (normal range, <9.0 U/mL). Histopathological examination of the erythema and bullae revealed subepidermal blistering accompanied by eosinophilic infiltration (Figure 2A). Direct immunofluorescence assay revealed immunoglobulin G deposition in the basement membrane zone (Figure 2B). Bullous pemphigoid was diagnosed. Erythema and bullae improved after administration of high‐dose systemic corticosteroid.

We furthermore approached the phenomenon of the distribution of the active lesion away from the inactive lesion by immunohistochemistry. We counted CD4^+^, CD25^+^, and FoxP3^+^ cells in the width of 4 mm of the tissue obtained from active lesion, inactive lesion, and intact skin (Table 1, Figure 2C‐E). Compared to the intact skin, CD4^+^ cells were counted 2.22‐fold and 1.83‐fold in active lesion and inactive lesion, respectively. Similarly, CD25^+^ cells were counted 1.48‐fold and 2.33‐fold. FoxP3^+^ cells were counted 1.75‐fold and 2.64‐fold.

**TABLE 1:** Number of infiltrating cells characterized by surface phenotype

Lesion	CD4	CD25	FoxP3
Active lesion	282	59	77
Inactive lesion	233	93	116
Intact skin	127	40	44

## 3 DISCUSSION

This case was characterized by the peculiar distribution pattern for active and inactive lesions of BP, which were clearly separated from each other. This may be regarded as an example of *locus maioris resitentiae*, indicating a site of the body that offers resistance to onset of a disease.^2^ In general, the concept of the phenomenon of antigenic competition has been referred to when the effects of a second vaccination may be reduced by an unrelated vaccination provided simultaneously or just shortly beforehand. Our case differs from such typical presentations, since the responsible antigen was the same.

Although information is limited regarding skin disorders, this phenomenon was already documented in the early 1970s in the field of contact hypersensitivity. Kimber et al suggested that dendritic cells may play important roles in antigenic competition between two different antigens.^1^ Dearman et al^3^ suggested the involvement of reduced secretion of interleukin‐6 from dendritic cells in the lymph nodes might explain this phenomenon.

Haeberle et al reported that regulatory T‐cell (Treg) deficiency may induce pathogenic autoantibody reacting to 230‐kD bullous pemphigoid antigen, leading to the development of autoimmune bullous disease.^4^ Rosenblum et al^5^ demonstrated that skin‐resident memory Treg contribute to mitigating skin inflammation upon repeated antigen exposure. These reports altogether suggest that skin‐resident memory Treg located in the area of the pre‐existing eruption may be responsible for preventing the emergence of subsequent eruptions; therefore, we attempted to identify Treg in the upper dermis of active and inactive lesions. CD4^+^ cells, potentially including inflammation‐promoting cells and inflammation‐inhibiting cells, infiltrated more in the active lesion than in the inactive lesion (Table 1). Conversely, CD25^+^ and FoxP3^+^ cells, mainly including Treg, infiltrated more in the inactive lesion than in the active lesion (Figure 2C‐E). Compatible with the previous reports, our data suggest that Treg in the pre‐existing lesion may inhibit the expansion of active lesion, resulting in the antigenic competition‐like phenomenon in BP.

## CONFLICT OF INTEREST

The authors have no conflicts of interest to declare.

## AUTHOR CONTRIBUTIONS

MF: contributed to data curation and validation. YN: contributed to concertation and project administration. HS: contributed to resources. AA: contributed to supervision.
